# Autologous collagen-induced chondrogenesis with high tibial osteotomy for large collapsed steroid-induced osteonecrosis in a patient with systemic lupus erythematosus: a case report and literature review

**DOI:** 10.1186/s12891-025-09020-0

**Published:** 2025-07-28

**Authors:** Dong Hwan Lee, Bo Seung Bae, Seon Ae Kim, Mi‑La Cho, Seok Jung Kim

**Affiliations:** 1 Department of Orthopedic Surgery, Yeouido St. Mary’s Hospital, College of Medicine, The Catholic University of Korea, Seoul, Republic of Korea; 2 Department of Orthopedic Surgery, Uijeongbu St. Mary’s Hospital, College of Medicine, The Catholic University of Korea, Cheonbo-ro, Uijeongbu-si, Gyeonggi-do 271 11765 Republic of Korea; 3 Department of Pathology, College of Medicine, The Catholic University of Korea, Seoul, 06591 Republic of Korea

**Keywords:** Systemic lupus erythematosus, Secondary osteonecrosis, Steroid-induced osteonecrosis, Autologous collagen-induced chondrogenesis, Large osteochondral defect

## Abstract

**Background:**

Secondary osteonecrosis (ON) of the knee poses a treatment challenge, especially in young patients with systemic lupus erythematosus (SLE) requiring ongoing steroid therapy. Joint-preserving options for large osteochondral defects are limited, and there are no standardized protocols.

**Case presentation:**

We report the case of a 37-year-old female with a history of SLE and prior left total knee arthroplasty who presented with severe right knee pain and a progressive varus deformity. Imaging revealed a 4 × 2 cm osteochondral defect of the medial femoral condyle with over 10 mm depth and an 8° varus alignment. Given the patient’s young age and preference to avoid arthroplasty, we performed a combined medial opening high tibial osteotomy (HTO) and autologous collagen-induced chondrogenesis (ACIC) with iliac crest bone grafting. The procedure involved creation of large channels for graft placement and multiple drillings, followed by atelocollagen mixture gel application to enhance cartilage regeneration. The patient experienced progressive pain relief and functional improvement, achieving full weight bearing by 3 months. However, crutch-assisted ambulation was maintained until 6 months to reduce loading on the joint. Radiographs at 3, 9, and 18 months demonstrated gradual medial joint space widening. At 2-year follow-up, arthroscopic examination confirmed complete defect coverage with regenerated cartilage. No major complications occurred.

**Conclusion:**

Medial opening HTO combined with ACIC and autologous bone grafting may offer a feasible single-stage, joint-preserving solution for large osteochondral defects in steroid-induced secondary ON, expanding treatment options for young SLE patients.

## Introduction

Secondary osteonecrosis (ON) is an uncommon condition with challenging treatment options, and no standardized protocol has yet been established. Secondary ON is relatively common in patients with systemic lupus erythematosus (SLE), with reported incidence rates varying up to approximately 5%, and the risk of development is known to increase with higher steroid dosages [1–3]. Various approaches have been attempted for treating osteochondral defects caused by secondary ON, but outcomes remain inferior compared to those for typical cartilage lesions [4, 5]. In patients with SLE, who often cannot discontinue steroid therapy, recovery is frequently poor or significantly delayed despite various treatment modalities. Particularly in cases with large osteochondral defects, conservative management rarely provides symptomatic relief, necessitating surgical intervention.

While total knee arthroplasty (TKA) has been widely reported as a treatment option, many affected patients are young, creating a demand for joint-preserving surgery. Several approaches have been reported, including allogenic osteochondral grafting, autologous chondrocyte implantation (ACI), and osteochondral autograft transplantation (OATS) [6–8]. However, each approach has distinct advantages and limitations, making it difficult to establish a clear gold standard for these complex cases.

In this case, we performed Autologous Collagen-Induced Chondrogenesis (ACIC), a single-stage cartilage regeneration technique using atelocollagen, combined with autologous bone grafting and high tibial osteotomy (HTO) to treat a large osteochondral defect resulting from steroid-induced secondary ON in a patient with SLE [9, 10]. HTO was performed to offload the affected area through alignment correction, while ACIC was selected as a cost-effective procedure for cartilage regeneration of the defect. To our knowledge, no previous reports have documented the use of atelocollagen-based cartilage regeneration procedures with HTO in patients with secondary ON. Through this case report, we aim to present ACIC as a viable joint-preserving surgical option for large osteochondral defects in secondary ON.

## Case presentation

### Patient's history

A 37-year-old female presented to our clinic with right knee pain. She had been diagnosed with systemic lupus erythematosus (SLE) in 2007 and had been on continuous steroid treatment. In July 2008, she developed secondary osteonecrosis (ON) of the left medial femoral condyle with extensive involvement of the metaphysis, for which she had undergone total knee arthroplasty (TKA) by another surgeon. She had been functioning well with the TKA, and radiographs obtained in January 2021 showed no evidence of loosening or other abnormalities.

**Fig. 1:**
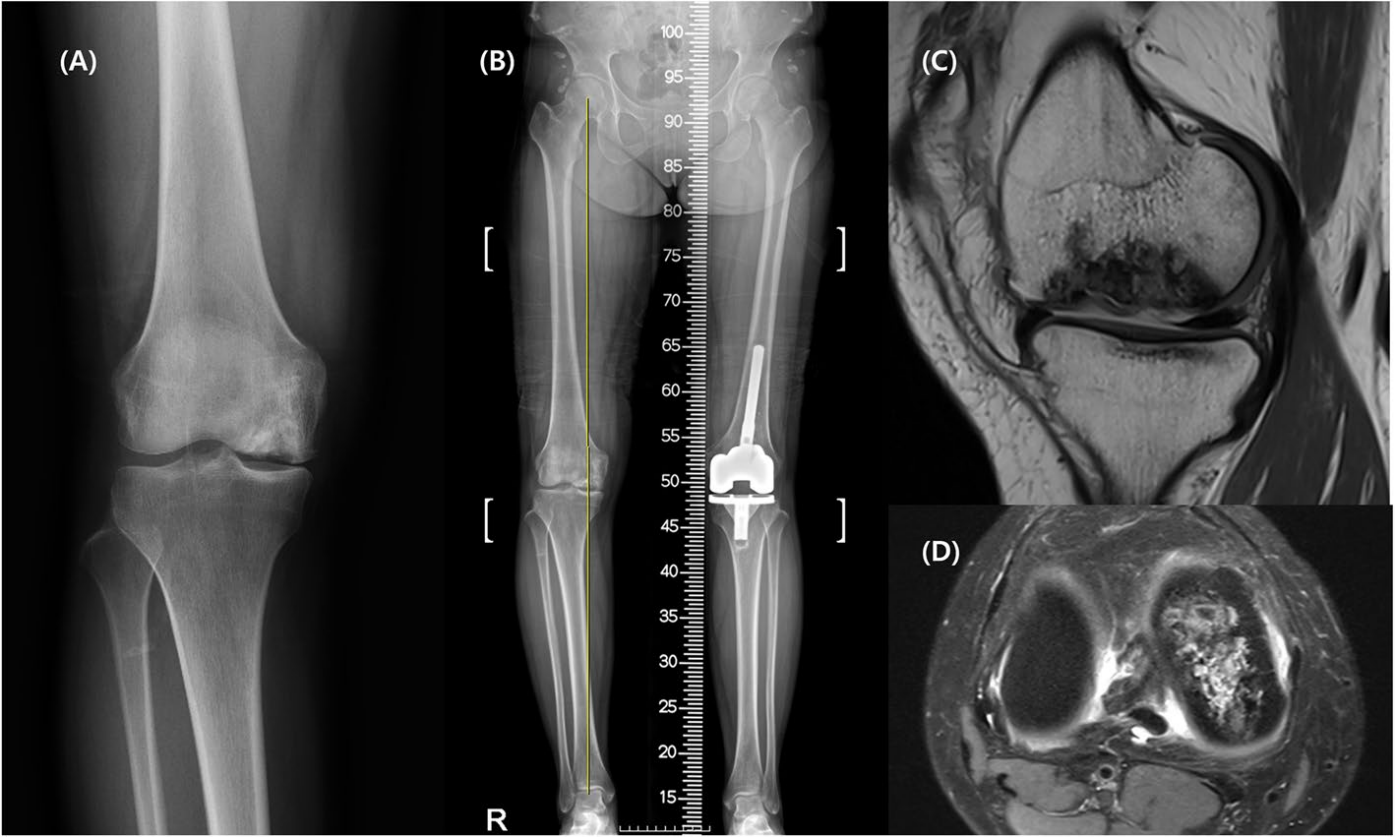
Preoperative imaging studies. **A** Anteroposterior radiograph of right knee. **B** Standing scanogram of whole lower limb demonstrating 8-degree varus alignment in the hip-knee-ankle axis. **C** Sagittal and (**D**) Axial magnetic resonance imaging of right knee showing large-sized collapsed osteonecrosis of the medial femoral condyle

The patient had experienced persistent mild pain in her right knee, which had recently become significantly worse with progressive varus deformity. At presentation, she reported pain severe enough to impede walking. Joint effusion was present, and range of motion (ROM) was limited to 0–90 degrees due to pain. At that time, SLE disease activity was assessed as low disease activity using the Systemic Lupus Erythematosus Disease Activity Index 2000 (SLEDAI-2 K), and the patient was maintained on prednisolone 5 mg once daily. Additionally, she was taking hydroxychloroquine 300 mg and celecoxib 200 mg once daily. Radiographic and MRI evaluation revealed secondary osteonecrosis with a large osteochondral defect of the medial femoral condyle and varus deformity (Fig. 1). Due to residual discomfort from her previous left knee surgery, the patient expressed a strong preference for joint-preserving surgery rather than arthroplasty. While allogenic osteochondral grafting might have been appropriate considering the defect's size and location, such allografts were unavailable in South Korea at the time of treatment. Alternative approaches were therefore necessary, and ACI was similarly unavailable when this surgery was performed. After thorough discussion and considering her young age and preferences, we planned a combined approach with medial opening high tibial osteotomy (HTO) for realignment and Autologous Collagen-Induced Chondrogenesis (ACIC) with bone grafting for cartilage regeneration.

### Preoperative planning and operative procedure

Preoperative standing lower extremity radiographs demonstrated an 8-degree varus alignment in the hip-knee-ankle (HKA) axis. The medial proximal tibial angle (MPTA) measured 84 degrees, and the mechanical lateral distal femoral angle (mLDFA) was 88 degrees. MRI revealed a large necrotic lesion measuring 4 × 2 cm with a depth exceeding 10 mm. Our surgical approach addressed two key issues: realignment and restoration of the necrotic lesion.

For realignment, we selected a medial opening HTO since the primary cause of varus alignment was tibial and the lesion was confined to the medial femoral condyle. We established a slightly more valgus correction target than usual, aiming for 65% of the Fujisawa point (normal target: 62.5%) to further offload the medial compartment given the extensive defect. The osteotomy gap size was 11 mm. Intraoperative imaging confirmed that proper correction to the target point was achieved. Fixation was accomplished using a fixed angle plate (Ohtofix plate, Ohtomedical Co. Ltd., Goyang, Korea) with locking screws.

**Fig. 2:**
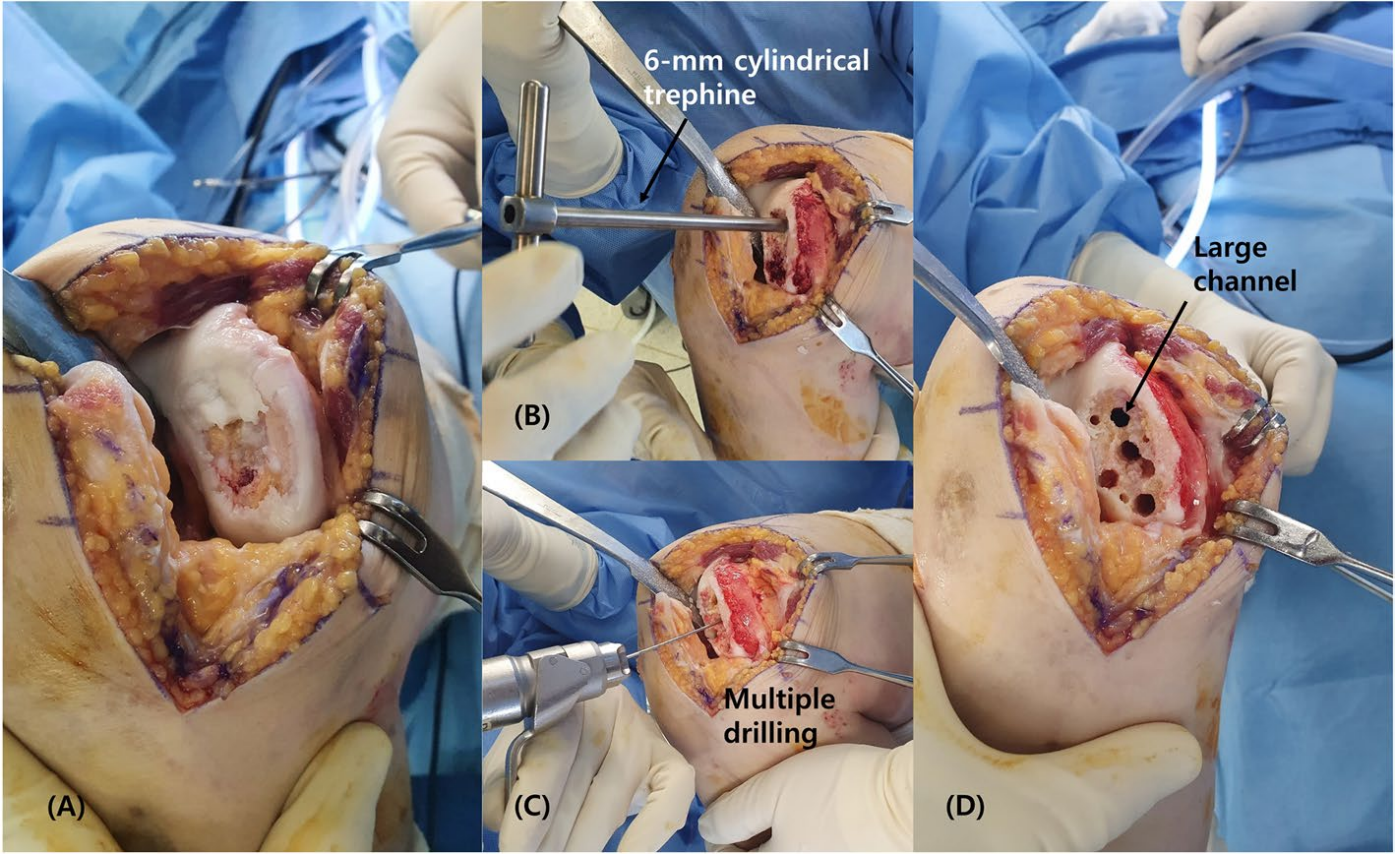
Intraoperative photographs. **A** Osteochondral defect measuring 4 × 2 cm^2^ within the osteonecrosis lesion. **B** Creation of large channels for bone grafting. **C** Formation of additional small channels through multiple drilling. **D** Completed preparation of the lesion

**Fig. 3:**
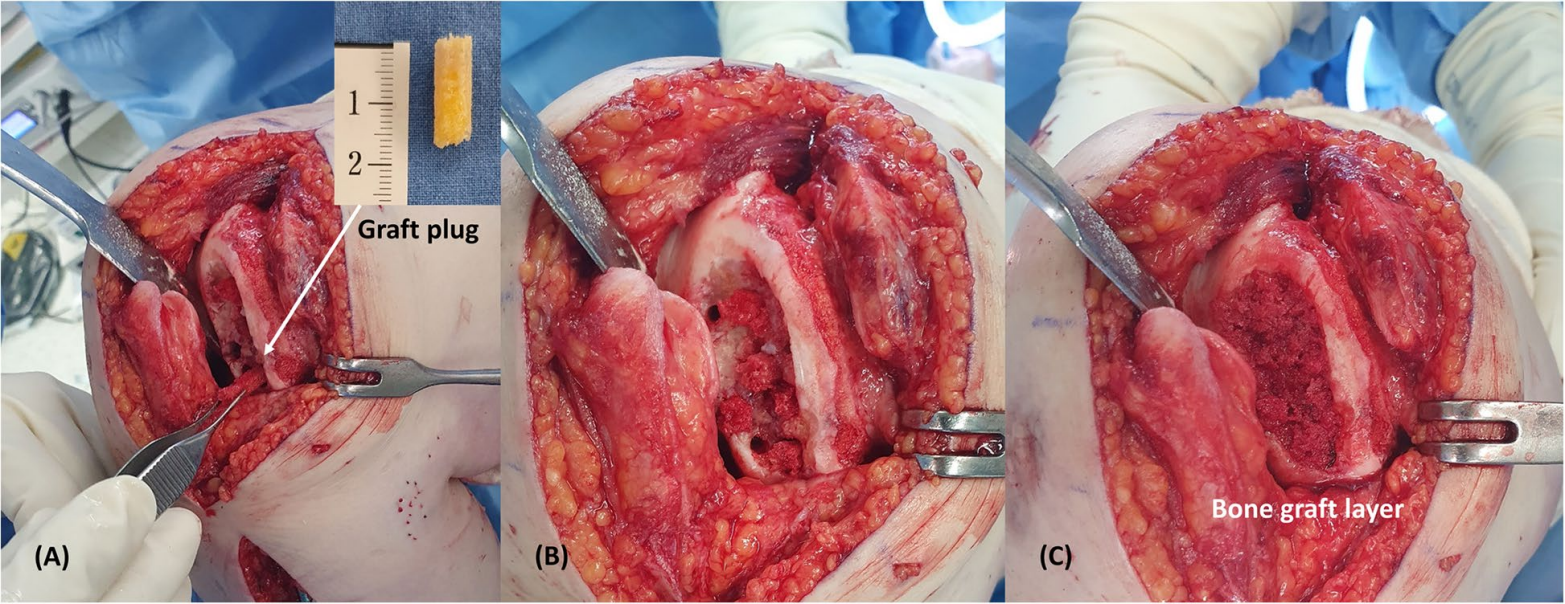
**A** Placement of prepared autologous bone donor into large channels. **B** Completed bone grafting of all large channels. **C** Subsequent bone grafting of the entire defect area

**Fig. 4:**
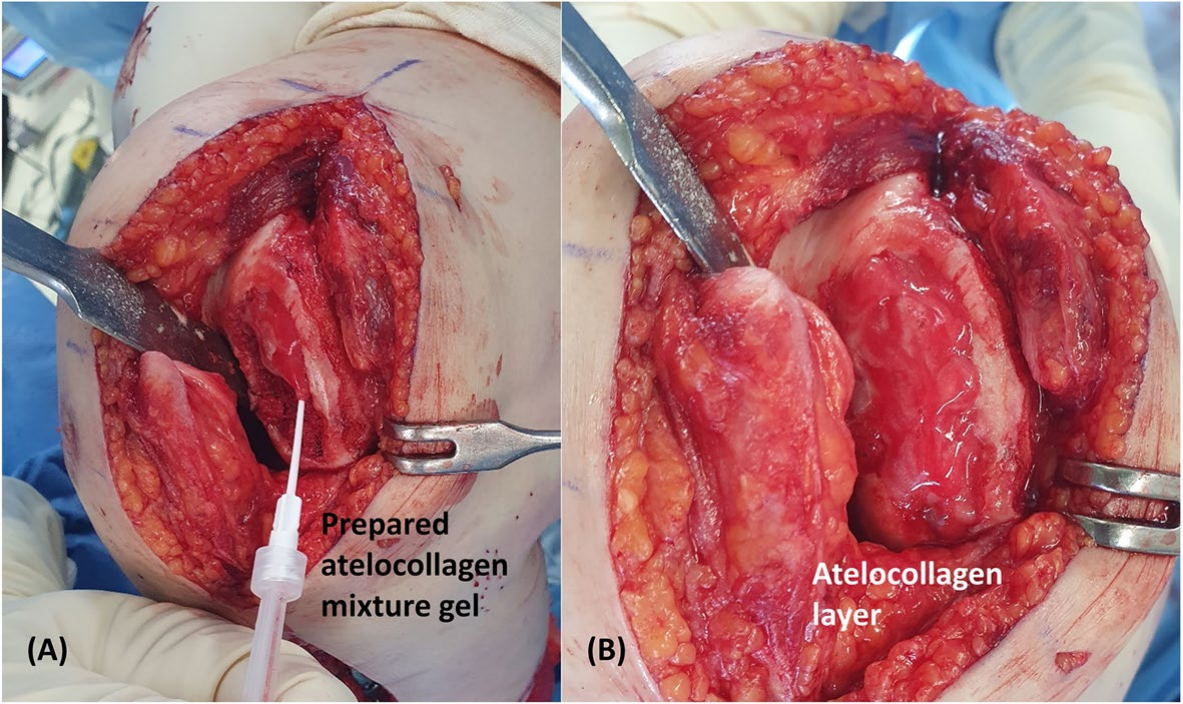
**A** Performing Autologous Collagen-Induced Chondrogenesis (ACIC) over the bone grafting area. **B** Complete coverage of the defect area with ACIC procedure

For the necrotic lesion restoration, we planned bone grafting followed by ACIC using atelocollagen, inspired by the"sandwich technique"used in ACI for large defects [11]. The necrotic lesion extended to depths of 10–15 mm over a 4 × 2 cm area, rendering complete debridement impractical. We first debrided the defect portion of the necrotic lesion and created channels to connect the remaining portion with normal bone marrow proximally. Using 6-mm cylindrical trephine, we created large channels for bone grafting, supplemented by multiple drilling with Kirschner wires (Fig. 2). About 10 cc of bone was harvested from the anterior iliac crest. Manual harvesting was conducted utilizing a 6-mm cylindrical trephine and bone harvester, resulting in six cylindrical graft plugs with a diameter of 6 mm and a length of 15 mm, which were subsequently transplanted into the prepared large channels within the necrotic area. The entire area excluding the channels was grafted with the remaining harvested bone (Fig. 3). Next, the atelocollagen mixture gel was prepared. Two 1 mL syringes and a Y-shaped mixing catheter were used. One syringe was filled with 1 mL of fibrinogen (Tisseel; Baxter, Thetford, United Kingdom), and the other syringe was filled with 0.9 mL of atelocollagen (COLTRIX® CartiRegen, Ubiosis Co. Ltd., Seongnam, Korea) and 0.1 mL of thrombin (50 IU). According to the manufacturers'information, COLTRIX® CartiRegen contains 3% type I atelocollagen, the fibrinogen solution contains 72–110 mg/mL of fibrinogen, and the thrombin solution contains 500 IU/mL; therefore, 0.1 mL of thrombin corresponds to 50 IU [9, 10]. The contents of both syringes were mixed using the Y-shaped catheter while being injected into the lesion. The atelocollagen mixture gel was meticulously applied over the bone-grafted lesion, and the gel typically solidified within approximately 5 min, preventing it from flowing away (Fig. 4).

### Postoperative course

The patient began gentle range of motion exercises on the second postoperative day, using continuous passive motion (CPM) with gradually increasing angles. She was initially non-weight bearing with crutch assistance, progressing to less than 50% partial weight bearing by 8 weeks, with gradual increases thereafter. Though full weight bearing was possible at 3 months, we advised continued crutch use to reduce load on the operated knee for a total of 6 months postoperatively. The patient initially used a removable splint, transitioning to a knee brace providing valgus force once swelling had subsided. The brace allowed range of motion and was maintained until 6 months postoperatively. For range of motion, we limited ROM to 90 degrees or less for only 2 weeks until swelling and pain improved, and thereafter proceeded with rehabilitation to rapidly increase ROM without restrictions.

This extended period of partial weight bearing and bracing was implemented due to the extensive lesion and the patient's underlying SLE. Patient compliance was excellent, with good adherence to rehabilitation protocols. No major complications occurred, though superficial wound dehiscence developed as a minor complication, which resolved after reducing the steroid dosage.

**Fig. 5:**
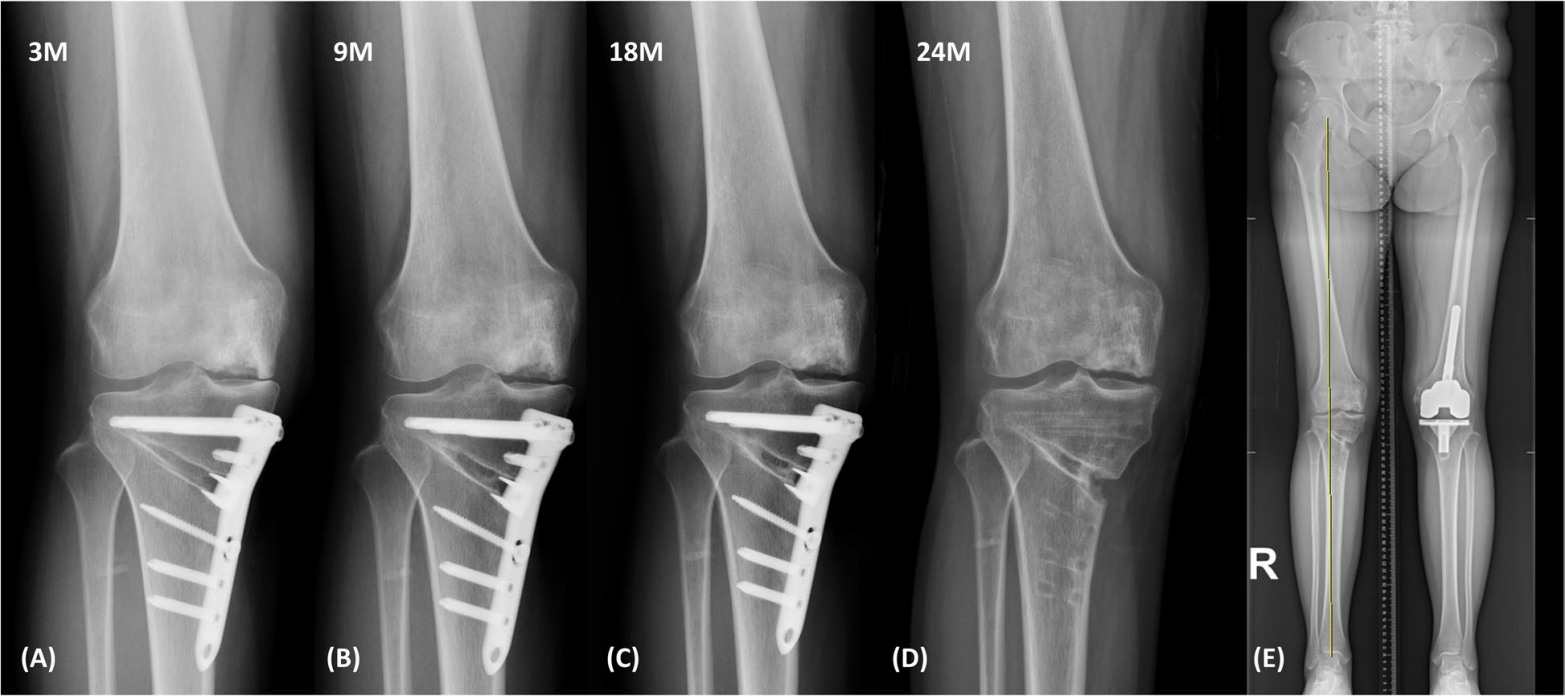
Standing anteroposterior radiograph of right knee after surgery: **A** 3 months, (**B**) 9 months, (**C**) 18 months, (**D**) 2 years. Progressive increase in medial joint space width is observed over time. Joint space width was 1 mm at 3 months, 1.5 mm at 9 months, 2.3 mm at 18 months, and 2.7 mm at 24 months postoperatively. **E** Scanogram of whole lower limb after metal plate removal at 2 years

**Fig. 6:**
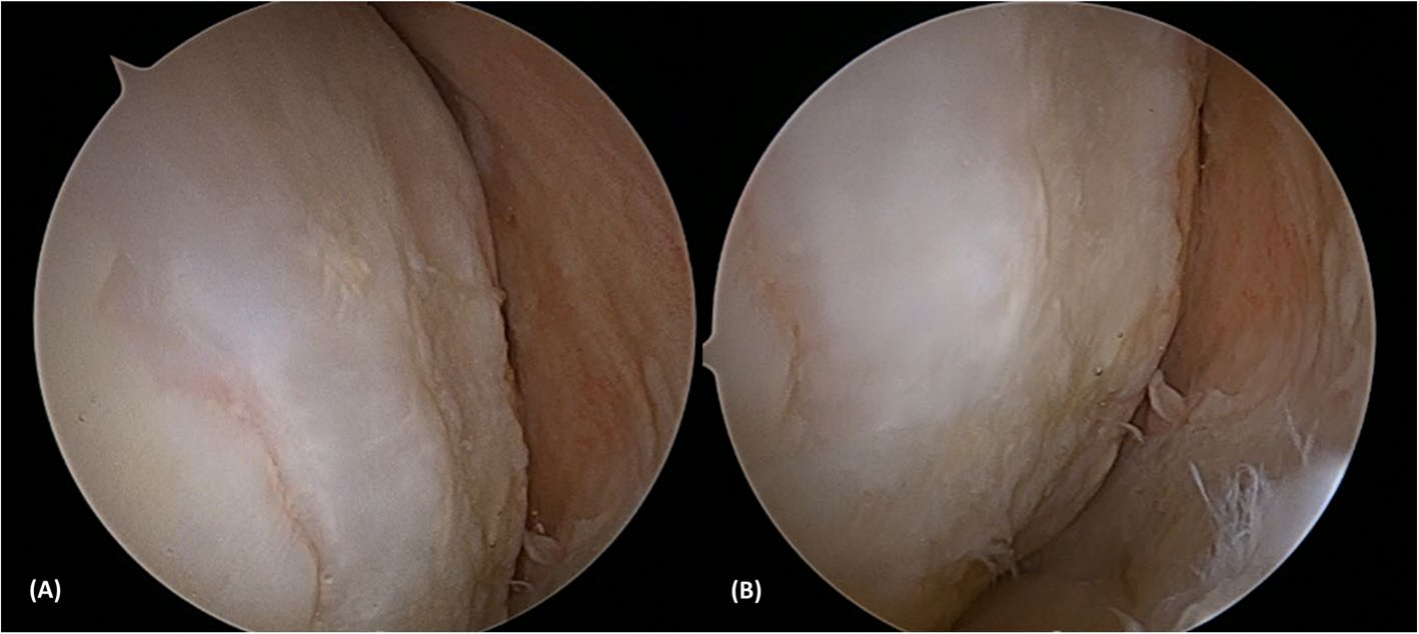
Arthroscopic images obtained 2 years postoperatively showing successful cartilage regeneration with appropriate congruency

Clinically, the patient reported medial pain for up to 2 months postoperatively, with significant improvement by 3 months. Joint effusion gradually decreased. By 9 months, symptoms had largely resolved, and at 1 year, she reported only minimal discomfort. The patient attained a pain-free full range of motion and expressed high satisfaction with the improvement in symptoms, including pain and effusion. Serial standing knee radiographs demonstrated progressive widening of the medial joint space (Fig. 5), suggesting successful regeneration. The joint space width was assessed by measuring the minimal distance between the femoral condyle and the tibial plateau in the medial compartment on standardized weight-bearing anteroposterior radiographs of the knee. At 2 years postoperatively, HKA alignment was 4° valgus, with adequate union at the osteotomy site, we performed metal plate removal and arthroscopic examination, which confirmed complete coverage of the previous large defect with regenerated cartilage (Fig. 6). The cartilage regeneration status evaluated through arthroscopic examination showed excellent improvement with Grade II on the International Cartilage Repair Society (ICRS) score. VAS, WOMAC, and Lysholm knee scores collected preoperatively and at 9 months and 2 years postoperatively also demonstrated excellent improvement (Table 1).

**Table 1:** Pre- and postoperative patient-reported outcome measures (PROMs)

Time point	VAS^*^ (0–10)	WOMAC^†^ (0–96)	Lysholm knee score^‡^ (0–100)
Preoperative	8	61	4
Postoperative (9 months)	3	23	68
Postoperative (2 years)	1	16	76

^*^Visual Analogue Scale for pain (VAS) (0–10; higher = worse pain)

^†^Western Ontario and McMaster Universities Osteoarthritis Index (WOMAC) (0–96; higher = worse symptoms)

^‡^Lysholm Knee Score (0–100; higher = better function)

## Discussion

Secondary osteonecrosis (ON) is considered to be an uncommon condition with challenges in treatment, and as such, a standardized treatment protocol has not yet been established. Steroid-induced and alcohol abuse have been identified as major causative factors of secondary ON [12, 13]. Among these, steroid-induced secondary ON often coexists with medical conditions requiring steroid use, making treatment even more difficult [5]. In particular, patients with Systemic Lupus Erythematosus (SLE) are often younger, have higher demands for treatment, and frequently need to maintain steroid use, which complicates their therapeutic management [4, 14]. Until collapse progresses, or in cases where the collapsed lesion is small, symptomatic treatment is maintained through medication and injection [15]. Recently, with advancements in cell therapy research, stem cell-based treatments like Bone Marrow Aspiration Concentrate (BMAC) have also been reported to yield favorable outcomes for small lesions [16–18].

However, for cases where the size of the collapsed lesion is large, these conservative treatments may not be effective. Surgical treatment becomes necessary at this point, yet there does not appear to be an established treatment protocol. Traditionally, arthroplasty has been widely used as a treatment for such large osteochondral defects in the knee [13, 19]. However, considering the patient's young age and underlying medical conditions, performing arthroplasty requires caution, and patients themselves often express resistance towards arthroplasty. Consequently, in accordance with these patient circumstances, there seems to be an increasing demand for and implementation of joint preserving surgery. For secondary ON with large osteochondral defects, the first joint preserving surgery to consider is allogenic osteochondral grafting [6, 20, 21]. Other reported options include microfracture alone, osteochondral autograft transplantation (OATS), and autologous chondrocyte implantation (ACI) [7, 8, 22]. Nishitani et al. reported positive long-term outcomes when performing OAT for osteochondral defects in steroid induced ON [8]. However, they noted a clear disadvantage of donor site morbidity, with their study confirming deterioration of the patellofemoral joint's Kellgren-Lawrence grade at final follow-up. Aydın et al. compared results after performing ACI in patients with secondary ON and osteochondritis dissecans (OCD) [7]. Although the therapeutic effect was inferior compared to the OCD group, good results with ACI were still observed in the secondary ON group. Nevertheless, ACI clearly has disadvantages including its high cost and requirement for a two-stage procedure. To facilitate comparison among these techniques, we compiled a comparative analysis of OCA, OATS, ACI, and the present case in Table 2.

**Table 2:** Joint-preserving options for large osteochondral defects in secondary osteonecrosis of the knee*

Technique	Defect size	Numbers	Outcomes	Limitations
Osteochondral allograft transplantation (OCA)	> 10 cm^2^	28 knees (Görtz 2010)	67 mo follow up; IKDC‑pain 7.1 → 2.0; IKDC‑function 3.5 → 8.3; KS‑function 60 → 85.7; graft survivorship 89%; only 1/28 converted to TKA	allograft cost & availability; tissue‑bank logistics; risk of immune problem
		33 knees (Early 2021)	Graft survivorship 90% at 5 y, 82% at 10 y; IKDC‑total 37.6 → 72.6; KOOS‑Pain 61.1 → 82.5; KOOS‑QoL 19.1 → 60.4; 85% (28/33) avoided TKA	
Osteochondral autograft transplantation (OATs)	mean 6.9 cm^2^ (range 5.3–8.5 cm^2^)	10 knees (Nishitani 2021)	IKDC 33 → 74 at 14‑y; no revisions	donor‑site morbidity; limited graft volume; requires specialized instrumentation
Autologous chondrocyte implantation (ACI)	mean 7.1 cm^2^ (range 4.2–10.5 cm^2^); mean depth 1.18 cm	21 knees (Aydın 2020)	ICRS 28 → 71; MRI cartilage thickness 1.4 mm at 5 y	two stages procedure; higher cost
ACIC (present case)	8 cm^2^ (4 × 2 cm), depth > 10 mm	1 knee	VAS 8 → 1; WOMAC 61 → 16; Lysholm 4 → 76 at 2y	single‑case data; short follow up period

^*^Abbreviations: *IKDC* International Knee Documentation Committee, *KS* Knee Society, *TKA* total knee arthroplasty, *KOOS* Knee injury and Osteoarthritis Outcome Score, *ICRS* International Cartilage Repair Society, *ACIC* autologous collagen‑induced chondrogenesis, *HTO* high tibial osteotomy, *VAS* Visual Analogue Scale, *WOMAC* Western Ontario and McMaster Universities Osteoarthritis Index

Autologous Collagen-Induced Chondrogenesis (ACIC) is a cartilage restoration procedure introduced by A.A. Shetty and S.J. Kim, which is performed as a single-stage operation using a gel-type collagen scaffold in an enhanced microfracture approach [9, 10]. As a relatively cost-effective procedure, its advantages over ACI are evident. In terms of cost comparison with other techniques, ACIC requires approximately $2,000 including surgical costs and materials, while ACI costs approximately $20,000 and OCA costs more than $10,000, varying with the allograft source. When comparing total treatment costs, microfracture has been reported at $24,000, OATS at $32,000, OCA at $75,000, and ACI at approximately $65,000 [23]. For ACIC, only the atelocollagen cost is added to the microfracture treatment cost, resulting in an estimated cost of $25,000–30,000, which is highly cost-effective. The drawbacks of OAT, such as donor site morbidity and size limitations, do not exist with ACIC. Considering these advantages compared to other techniques and the proven efficacy of ACIC in previous studies, surgery was performed under the judgment that this was a cost-effective technique suitable for this patient, and successful results have been demonstrated up to 2 years of follow-up. The basic science of ACIC involves increasing the chondrogenic differentiation of bone marrow mesenchymal stem cells (BM-MSCs) using atelocollagen. An atelocollagen and fibrin glue mixture is applied to the lesion, allowing BM-MSCs flowing from multiple drilling channels to interact with the applied atelocollagen. Therefore, when performing this technique in cases of secondary ON like the present case, it is important to establish sufficient channels with normal bone marrow. In this case, the defect was significant enough to require bone grafting, and due to the aforementioned reasons, adequate channels were necessary. Thus, large channels were created to form a bone graft bridge, and additional multiple drilling was performed to allow sufficient BM-MSCs to flow into the lesion. Furthermore, through these large channels and drilling, the effects of core decompression and BM-MSCs action can be induced, and some degree of bone regeneration in the entire necrotic area can also be expected [24].

Additionally, as the patient showed varus deformity in alignment, a medial opening HTO was performed for correction. Reported cases of corrective osteotomy in SLE patients are rare. A case of medial closing distal femoral osteotomy has been reported, where autologous bone grafting was performed on the ON area and the cartilage was treated through repair [25]. While the corrective osteotomy method in that study differs from ours, considering the effective treatment in both studies, corrective osteotomy can be considered as a treatment option for SLE patients. However, the union rate may be lower or the frequency of other complications may be higher, necessitating further studies involving a larger number of SLE patients.

While questions remain about cartilage quality after ACIC treatment, the six-year clinical outcomes are encouraging [9]. This technique overcomes the limitations of both ACI and OAT, making it a valuable treatment option. Our case demonstrates that ACIC can successfully address even large osteonecrotic lesions. Concurrent high tibial osteotomy (HTO) was performed due to the patient's varus deformity, facilitating offloading of the lesion site and promoting cartilage regeneration via ACIC, resulting in favorable-quality cartilage regeneration. Arthroscopic evaluation classified the repair tissue as ICRS Grade II, a level that generally represents a mixture of hyaline and fibrocartilage rather than purely hyaline cartilage, and this should be considered when interpreting long-term durability. Careful preparation of the defect site appears important to success, and we anticipate further positive results as more surgeons gain experience with this technique.

## Conclusion

In our case, an SLE patient with large osteonecrotic defect and varus deformity achieved satisfactory results through medial opening HTO with ACIC. To our knowledge, this represents the first application of atelocollagen scaffold for steroid-induced osteonecrosis, particularly for such a large defect. We propose that ACIC can be considered as one of the options for joint preserving surgery in secondary ON.

## Data Availability

All data concerning the case are presented in the manuscript.
